# Supplementary material for: The Chloroplast Genome of Endive (Cichorium endivia L.): Cultivar Structural Variants and Transcriptome Responses to Stress Due to Rain Extreme Events
Source: Genes (Basel). 2023 Sep 21;14(9):1829. doi: 10.3390/genes14091829 (PMC10531310; doi:10.3390/genes14091829)
Supplement: Supplementary file 1 [file genes-14-01829-s001.zip › Figure S1.pdf]

A)

B)

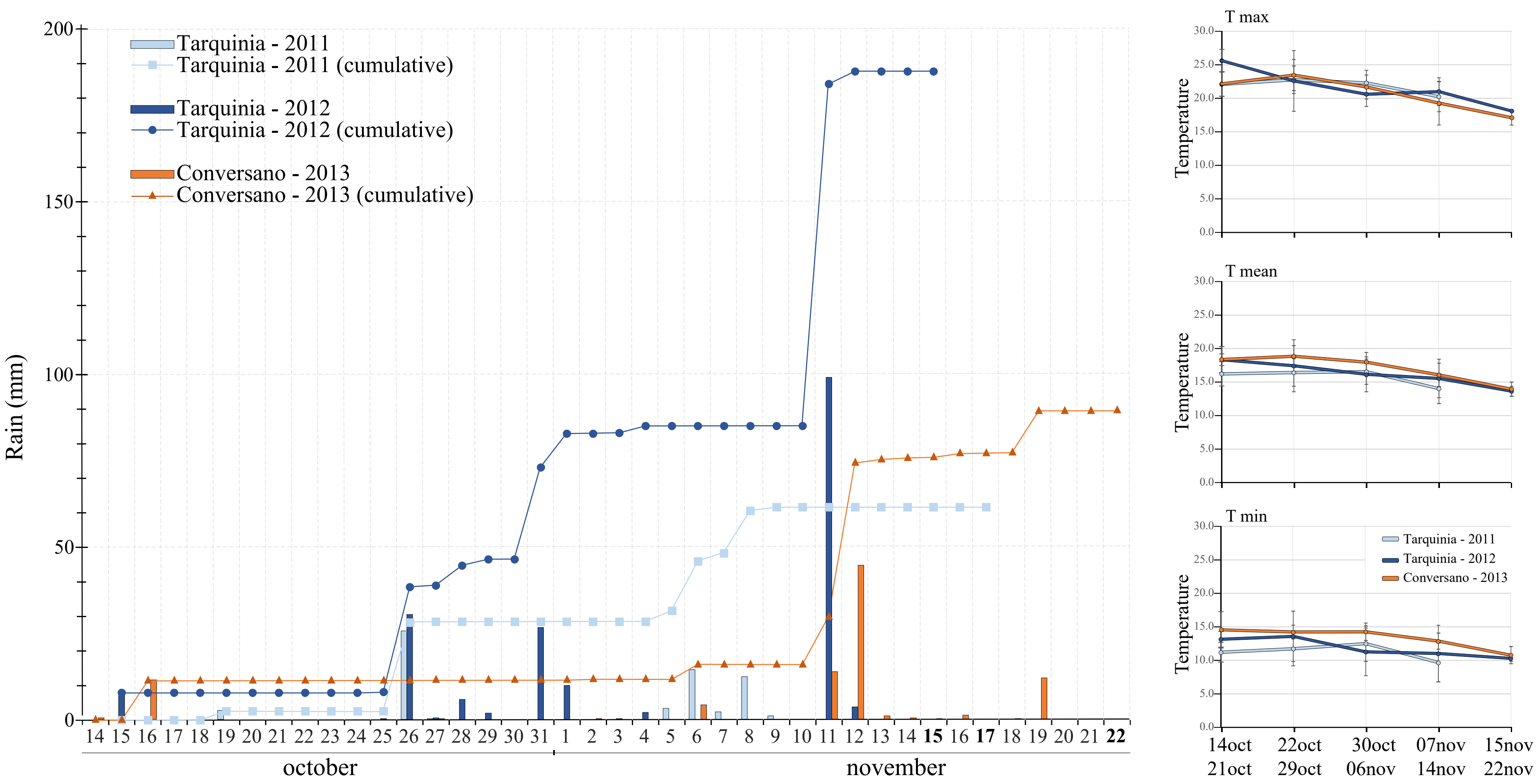

Figure S1 Climatic data related to cultivations in Tarquinia (2011 and 2012; Lazio, Italy) and Conversano (2013; Apulia, Italy).
A) Cumulative rainfall and rainy days (left y-axis) one month before harvest. **B**) Minimum, average and maximum temperature recored in the four weeks before harvest
