# Supplementary material for: The Chloroplast Genome of Endive (Cichorium endivia L.): Cultivar Structural Variants and Transcriptome Responses to Stress Due to Rain Extreme Events
Source: Genes (Basel). 2023 Sep 21;14(9):1829. doi: 10.3390/genes14091829 (PMC10531310; doi:10.3390/genes14091829)
Supplement: Supplementary file 1 [file genes-14-01829-s001.zip › Figure S2.pdf]

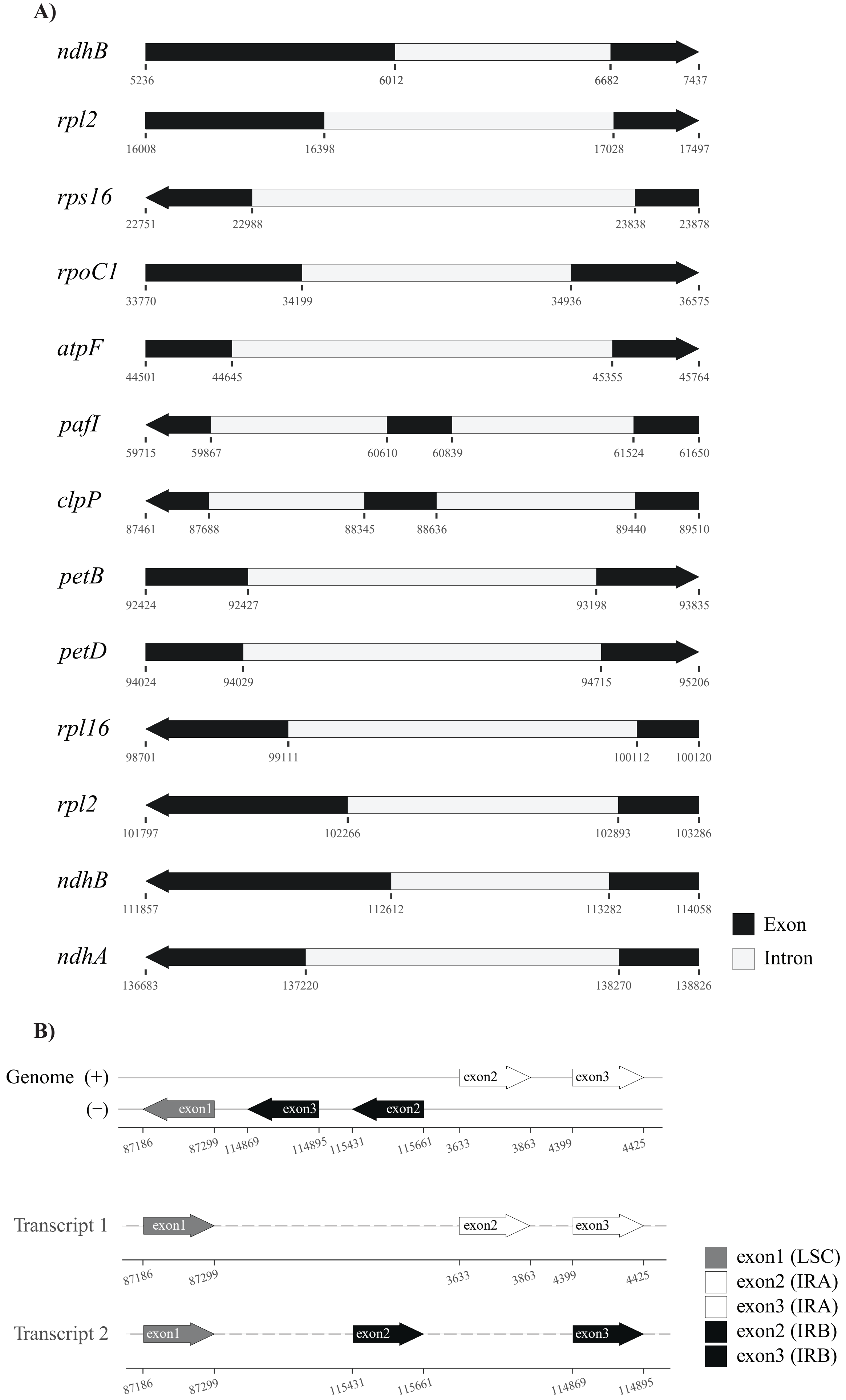

Figure S2. Splicing events in endive chloroplast genes. A) Cis-splicing genes. Genes are shown as oriented arrows with exons (dark boxes) and introns (white boxes). The numbers underneath mark the region boundaries. **B**) Trans-splicing of the *rps12* gene. Exons are reported as oriented arrows. Exon 1 was in the LSC, while exons 2 and 3 were in the IRs.
