# Supplementary material for: The Chloroplast Genome of Endive (Cichorium endivia L.): Cultivar Structural Variants and Transcriptome Responses to Stress Due to Rain Extreme Events
Source: Genes (Basel). 2023 Sep 21;14(9):1829. doi: 10.3390/genes14091829 (PMC10531310; doi:10.3390/genes14091829)
Supplement: Supplementary file 1 [file genes-14-01829-s001.zip › Figure S3.pdf]

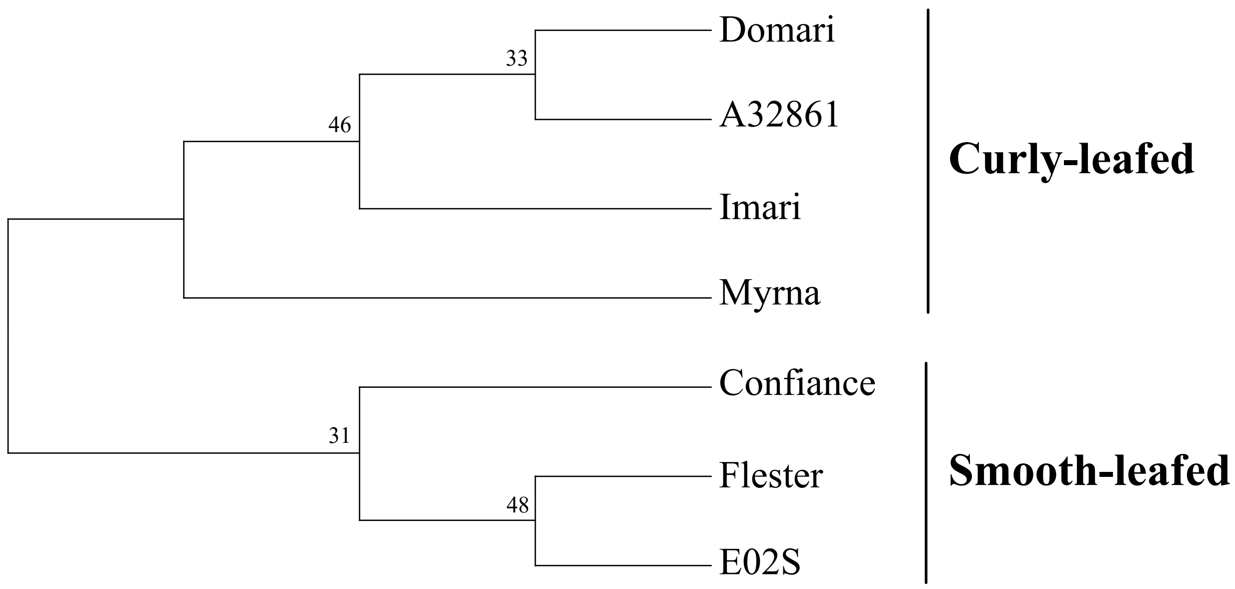

Figure S3. Phylogenetic tree from maximum-likelihood (ML) method based on concatenated SNP sequence alignment from the seven *C. endivia* cultivars. Numbers refer to ML bootstrap support values.
