# Supplementary material for: The Chloroplast Genome of Endive (Cichorium endivia L.): Cultivar Structural Variants and Transcriptome Responses to Stress Due to Rain Extreme Events
Source: Genes (Basel). 2023 Sep 21;14(9):1829. doi: 10.3390/genes14091829 (PMC10531310; doi:10.3390/genes14091829)
Supplement: Supplementary file 1 [file genes-14-01829-s001.zip › Table S1.pdf]

Table S1. *Month average values of rain, relative humidity and temperatures of the entire cycle (September-November)*

**Site – year (acronym)**	**Parameters**	**Sept**	**Oct**	**Nov**	**mean±sd**
**Tarquinia - 2011** (MRY)	T. min (°C)	15.53	11.74	10.51	12.6±2.6
	T. avg (°C)	22.15	17.41	14.85	18.1±3.7
	T. max (°C)	28.88	23.99	20.92	24.6±4.0
	Rain (mm)	0.36	0.92	**2.21**	1.2±0.9
	Cum. rain (mm)	4.38	12.28	54.95	23.9±27.2
	RH (%)	75.19	74.25	80.99	76.8±3.6
**Tarquinia - 2012** (HRY+WL)	T. min (°C)	17.04	13.58	11.32	14±2.9
	T. avg (°C)	22.28	18.36	15.79	18.8±3.3
	T. max (°C)	27.54	24.45	20.80	24.3±3.4
	Rain (mm)	0.40	3.41	6.74	3.5±3.2
	Cum. rain (mm)	0.71	38.88	164.86	68.2±85.9
	RH (%)	83.85	89.33	86.72	86.6±2.7
**Conversano - 2013** (HRY)	T. min (°C)	15.98	14.63	12.42	14.3±1.8
	T. avg (°C)	20.48	18.60	15.74	18.3±2.4
	T. max (°C)	24.98	22.57	19.07	22.2±3.0
	Rain (mm)	0.57	1.25	**3.55**	1.8±1.6
	Cum. rain (mm)	0.73	35.48	81.97	39.4±40.8
	RH (%)	64.83	77.13	78.32	73.4±7.5

MRY, moderately rainy year
HRY+WL, highly rainy year and waterlogging
HRY, highly rainy year
