# Supplementary material for: The Chloroplast Genome of Endive (Cichorium endivia L.): Cultivar Structural Variants and Transcriptome Responses to Stress Due to Rain Extreme Events
Source: Genes (Basel). 2023 Sep 21;14(9):1829. doi: 10.3390/genes14091829 (PMC10531310; doi:10.3390/genes14091829)
Supplement: Supplementary file 1 [file genes-14-01829-s001.zip › Table S3.pdf]

Table S3. *Nutrient content variation (mg g−1 dry weight) of leaves in year affected by heavy rain*

Compounds[a]	MRY[b]		HRY		Significance[c]		
	Domari	Flester	Domari	Flester	G	Y	GxY
αGLC	40.36±9.83	37.95±6.88	30.77±4.64	31.14±4.84	*n.s.*	*	*n.s.*
βGLC	77.06±17.56	72.13±13.67	59.83±10.4	58.28±8.91	*n.s.*	*	*n.s.*
FRU	148.21±35.64	142.64±26.08	115.68±11.12	114.86±17.94	*n.s.*	*	*n.s.*
INUL	2.59±0.42a	1.3±0.3b	1.4±0.59b	1.07±0.08b	***	***	**
KES	8.6±2.13	4.22±0.77	4.43±0.95	2.18±0.38	***	***	*n.s.*
SUC	34.65±5.97	22.53±2.22	27.9±2.91	20.77±2.82	***	**	*n.s.*
CAR-T	*311.47±64.11*	*280.77±47.75*	*147.94±19.14*	*136.88±19.95*	*n.s.*	***	*n.s.*
CA	3.91±1.41	4.46±1.15	3.33±0.61	5.41±1.08	*n.s.*	*n.s.*	*n.s.*
FA	0.09±0.03	0.11±0.02	0.07±0.01	0.07±0.01	*	*n.s.*	*n.s.*
LA	0.05±0.03	0.06±0.03	0.06±0.01	0.07±0.03	*n.s.*	*n.s.*	*n.s.*
MA	19.69±1.77	18.92±1.83	24.7±7.27	23.22±4.2	*n.s.*	*	*n.s.*
SA	0.19±0.06	0.29±0.06	0.37±0.05	0.45±0.06	***	***	*n.s.*
TA	1.29±0.27	0.94±0.21	0.8±0.37	0.66±0.12	**	**	*n.s.*
OA-T	*25.21±2.45*	*24.79±2.6*	*29.33±7.52*	*29.88±4.93*	*n.s.*	*n.s.*	*n.s.*
ALA	0.6±0.11	0.55±0.12	0.43±0.07	0.43±0.06	*n.s.*	**	*n.s.*
ASN	6.01±1.3	4.09±1.12	4.76±1.28	2.95±1.02	**	*n.s.*	*n.s.*
ASP	1.54±0.34a	1.39±0.19a	0.95±0.2b	1.26±0.14ab	*n.s.*	***	*
GABA	0.86±0.24	0.98±0.17	0.62±0.1	0.51±0.1	*n.s.*	*	*n.s.*
GLN	6.13±2.22	2.3±0.8	3.58±0.45	1.32±0.46	***	**	*n.s.*
GLU	2.69±0.85a	1.76±0.47c	1.87±0.17bc	2.14±0.19b	**	*	*
ILE	0.15±0.04ab	0.16±0.03ab	0.22±0.13a	0.13±0.03b	*n.s.*	*	*
PHE	0.13±0.04b	0.24±0.04a	0.11±0.05b	0.14±0.02b	***	*n.s.*	*
THR	0.45±0.13	0.31±0.05	0.45±0.15	0.24±0.07	**	*n.s.*	*n.s.*
VAL	0.27±0.07	0.3±0.03	0.23±0.12	0.17±0.02	*n.s.*	*n.s.*	*n.s.*
AA-T	*18.85±4.13*	*12.08±1.78*	*13.22±2.07*	*9.29±2.06*	***	**	*n.s.*
CHA	0.87±0.41c	1.16±0.68c	2.09±0.34b	3.55±0.79a	*n.s.*	***	*
MCTA	0.08±0.02	0.07±0.03	0.14±0.04	0.14±0.03	*n.s.*	**	*n.s.*
HCA-T	*0.95±0.42c*	*1.23±0.69c*	*2.23±0.38b*	*3.68±0.8a*	*n.s.*	***	*
CI	0.66±0.27	0.21±0.07	0.62±0.11	0.22±0.05	***	*n.s.*	*n.s.*
MI	5.76±0.58	5±0.39	3.83±0.64	3.4±0.55	**	***	*n.s.*
SI	0.19±0.05	0.45±0.09	0.16±0.05	0.39±0.09	***	*n.s.*	*n.s.*
QA	0.38±0.06	0.24±0.05	0.3±0.03	0.19±0.02	***	**	*n.s.*
POL-T	*7±0.88*	*5.89±0.5*	*4.91±0.68*	*4.2±0.54*	**	***	*n.s.*
CHN	1.2±0.11	1.3±0.09	1.23±0.18	1.36±0.08	*n.s.*	*n.s.*	*n.s.*
ETA	0.8±0.22	0.96±0.16	0.78±0.09	0.74±0.14	*n.s.*	*n.s.*	*n.s.*
OTR-T	*2.01±0.27*	*2.26±0.2*	*2.01±0.26*	*2.1±0.21*	*	*n.s.*	*n.s.*

a, Nutritive compounds. Carbohydrates: GLC, Glucose; FRU, Fructose; INUL, inulin; KES, Kestose; SUC, Sucrose; CAR-T, total carbohydrates. Organic acids: CA, Citric acid; FA, Fumaric acid; LA, Lactic acid; MA, Malic acid; SA, Succinic acid; TA, Tartaric acid; OA-T, total organic acids. Amino acids: ALA, Alanine; ASN, Asparagine; ASP, Aspartic acid; GABA, γ-Aminobutyric acid; GLN, Glutamine; GLU, Glutamic acid; ILE, Isoleucine; PHE, Phenylalanine; THR, Threonine; VAL, Valine; AA-T, total amino acids. Hydroxycinnamic acids: CHA, Chicoric acid; MCTA, Monocaffeoyl tartaric acid; HCA-T, total hydroxycinnamic acids. Polyols: CI, Chiro-inositol; MI, Myo-inositol; SI, Scyllo-inositol; QA, Quinic acid; POL-T, total polyols. Others: CHN, Choline; ETA, Ethanolamine; OTR-T, total other compounds.

b, MRY, moderately rainy year; HRY, highly rainy year.

c, G, genotype; Y, year; G × Y, genotype: year interaction. Significance letters refer to G × Y effect. *, **, *** = significant at $P \leq 0.05$, 0.01 and 0.001 respectively. n.s., non-significant.
